# Author Correction: NOTCH localizes to mitochondria through the TBC1D15-FIS1 interaction and is stabilized via blockade of E3 ligase and CDK8 recruitment to reprogram tumor-initiating cells

**DOI:** 10.1038/s12276-024-01325-9

**Published:** 2024-10-28

**Authors:** Hye Yeon Choi, Yicheng Zhu, Xuyao Zhao, Simran Mehta, Juan Carlos Hernandez, Jae-Jin Lee, Yi Kou, Risa Machida, Mauro Giacca, Giannino Del Sal, Ratna Ray, Hyungjin Eoh, Stanley M. Tahara, Lin Chen, Hidekazu Tsukamoto, Keigo Machida

**Affiliations:** 1 Departments of Molecular Microbiology and Immunology, University of Southern California, Los Angeles, CA USA; 2 Viterbi School of Engineering, University of Southern California, Los Angeles, CA USA; 3 International Centre for Genetic Engineering and Biotechnology, Trieste, Italy; 4 Department of Life Sciences, University of Trieste, 34127 Trieste, Italy; 5 International Centre for Genetic Engineering and Biotechnology (ICGEB), Area Science Park-Padriciano, Trieste, Italy; 6 IFOM ETS, The AIRC Institute of Molecular Oncology, Milan, Italy; 7 Saint Louis University, School of Medicine, St Louis, MO USA; 8 Department of Pathology, University of Southern California, Los Angeles, CA USA; 9 Southern California Research Center for ALPD and Cirrhosis, Los Angeles, CA USA

**Keywords:** Oncogenesis, Cancer stem cells

Correction to: *Experimental & Molecular Medicine* 10.1038/s12276-024-01174-6, published online 27 February 2024

We carelessly arranged a liver tissue image of control Western diet (WD)-fed *Notch1*^*FL/FL*^ *NS5A* transgenic mouse of Fig. 3e with incorrect image. The error was introduced during figure preparation and is not a correct representation of the data. The authors have provided a corrected Fig. 3e. The error in the original published manuscript does not alter the results or conclusions of this study. The authors apologize that these errors were not detected earlier. We also attached a revised panel of Figure of this paper. We sincerely apologize for this caused problem.

**Figure:**
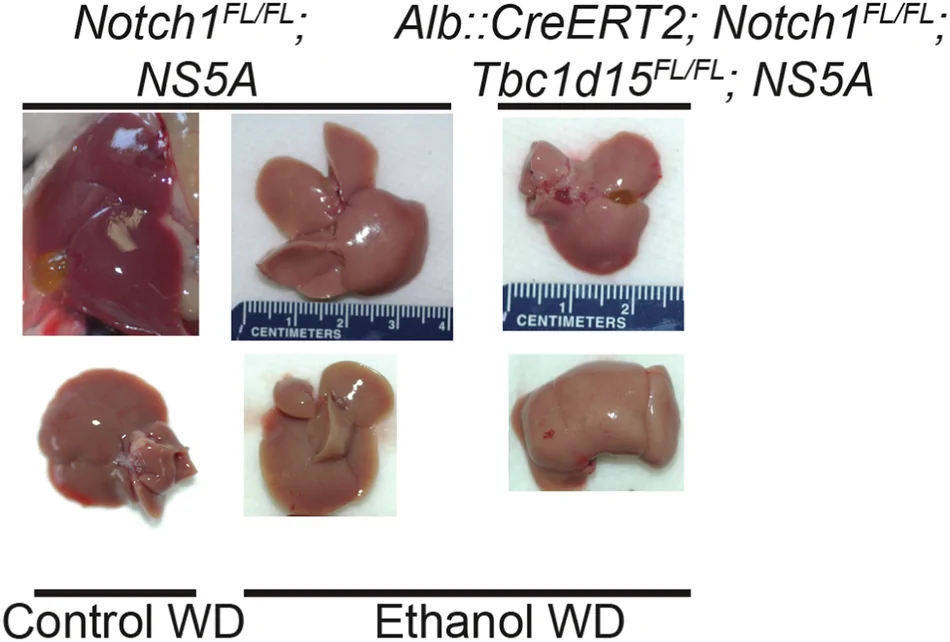

The original article has been corrected.

